# ﻿On eleven new species of the orb-weaver spider genus *Araneus* Clerck, 1757 (Araneae, Araneidae) from Xishuangbanna, Yunnan, China

**DOI:** 10.3897/zookeys.1137.96306

**Published:** 2022-12-22

**Authors:** Xiaoqi Mi, Shuqiang Li

**Affiliations:** 1 College of Agriculture and Forestry Engineering and Planning, Guizhou Provincial Key Laboratory of Biodiversity Conservation and Utilization in the Fanjing Mountain Region, Tongren University, Tongren 554300, Guizhou, China Tongren University Tongren China; 2 Institute of Zoology, Chinese Academy of Sciences, Beijing 100101, China Institute of Zoology, Chinese Academy of sciences Beijing China

**Keywords:** Arachnida, morphology, species, taxonomy, type

## Abstract

Eleven new species of *Araneus* Clerck, 1757 from Xishuangbanna, Yunnan, China are described: *Araneusarcuatus***sp. nov.** (♂♀), *A.bidentatus***sp. nov.** (♂♀), *A.bidentatoides***sp. nov.** (♂♀), *A.complanatus***sp. nov.** (♂♀), *A.corrugis***sp. nov.** (♀), *A.cucullatus***sp. nov.** (♀), *A.minisculus***sp. nov.** (♂), *A.ovoideus***sp. nov.** (♀), *A.pseudodigitatus***sp. nov.** (♂♀), *A.semiorbiculatus***sp. nov.** (♀), and *A.tetracanthus***sp. nov.** (♂♀). Diagnostic photographs of the habitus and copulatory organs are provided.

## ﻿Introduction

A large number of spider species live in the 1125 ha Xishuangbanna Tropical Botanical Garden (XTBG), including 782 spider species recorded through an “All Species Inventory” (Wang et al. 2020; Li et al. 2021; Yao et al. 2021; Liu et al. 2022; Lu et al. 2022; Zhao et al. 2022). The number of known species of the orb-weaver spider family Araneidae Clerck, 1757 in this region continues to increase with ongoing research (Mi and Li 2021a, b).

A total of 3092 species in 183 genera of Araneidae are known worldwide (WSC 2022), of which 402 species in 50 genera have been recorded from China (Li 2020). As the largest genus of the family, *Araneus* Clerck, 1757 includes 552 species worldwide, 342 of them are known only from a single sex or juveniles (WSC 2022). There are great differences in the habitus and copulatory organs among species, which suggests that *Araneus* may be polyphyletic. Scharff et al. (2020) found that 11 purported *Araneus* species from the Austral region belong instead to seven new genera.

In this study, 15 *Araneus* species from XTBG and the surrounding areas were identified, including *A.viridisomus* Gravely, 1921, *A.noegeatus* (Thorell, 1895), *A.nidus* Yin & Gong, 1996, *A.fengshanensis* Zhu & Song, 1994, and 11 new species. Even though the 11 species described here significantly differ, they are placed in *Araneus* provisionally until a phylogenetic analysis is conducted.

## ﻿Materials and methods

All of the specimens were collected by fogging, beating shrubs, or hand collecting and are preserved in 75% ethanol. Type specimens of the new species are deposited in the Institute of Zoology, Chinese Academy of Sciences (IZCAS) in Beijing. The specimens were examined with an Olympus SZ51 stereomicroscope. The epigynes were cleared in lactic acid for examination and imaging. The left male palps were dissected in ethanol for examination, description, and imaging. Photographs of the habitus and copulatory organs were taken with a Kuy Nice CCD mounted on an Olympus BX43 compound microscope. Compound focus images were generated using Helicon Focus v. 6.7.1.

All measurements are given in millimeters. Leg measurements are given as total length (femur, patella + tibia, metatarsus, tarsus). References to figures in the cited papers are listed in lowercase (fig. or figs); figures in this paper are noted with an initial capital (Fig. or Figs). Abbreviations used in the text and figures are as follows: ALE anterior lateral eye; AME anterior median eye; BE broken embolus; C conductor; CD copulatory duct; CO copulatory opening; E embolus; FD fertilization duct; H hood; MA median apophysis; MOA median ocular area; PLE posterior lateral eye; PME posterior median eye; Sc scape; Sp spermatheca; TA terminal apophysis; TE tegular extension.

## ﻿Taxonomy

### Family Araneidae Clerck, 1757

#### ﻿Genus Araneus

Taxon classificationAnimaliaAraneaeAraneidae

Clerck, 1757

8D7A977F-D67D-5900-8BE4-81692088BEF9

Araneus Clerck, 1757: 22.

##### Type species.

*Araneusangulatus* Clerck, 1757.

#### ﻿ Araneus arcuatus sp. nov.

Taxon classificationAnimaliaAraneaeAraneidae

5E26719A-DC1B-590B-BE97-9C41714AB40A

https://zoobank.org/9ABF17D1-1973-4170-BF40-694237E504CE

Figs 1 , 2 , 18A–D

##### Type material.

***Holotype*** ♂ (IZCAS-Ar43081), China: Yunnan, Xishuangbanna, Mengla County, Menglun Township, Menglun Nature Reserve, XTBG (21°54.18'N, 101°16.90'E, ca 610 m), 5.V.2019, Y.F. Tong leg. ***Paratypes***: 1♂ (IZCAS-Ar43082), same data as holotype; 1 ♀ (IZCAS-Ar43083), secondary tropical forest, bamboo plantation along G213 roadside (21°53.82'N, 101°16.99'E, ca 610 m), 3.VIII.2018, Z.L. Bai leg.; 1♂ (IZCAS-Ar43084), G213 roadside near Lüshilin (21°53.91'N, 101°17.01'E, ca 620 m), 12.VIII.2018, C. Wang leg.; 2♂2♀ (IZCAS-Ar43085–43088), Masuoxing Village (21°54.02'N, 101°16.90'E, ca 560 m), 27.IV.2019, Y.F. Tong leg.; 1♂ (IZCAS-Ar43089), #3 site around the dump (21°54.34'N, 101°16.79'E, ca 620 m), 2.V.2019, Y.F. Tong leg.; 1♀ (IZCAS-Ar43090), #6 site around the dump (21°54.33'N, 101°16.79'E, ca 620 m), 7.V.2019, Y.F. Tong leg. Other material examined: 1♂ (IZCAS-Ar43091), 55 km from Xishuangbanna National Nature Reserve, artificial *Ficusmicrocarpa* forest (21°54.97'N, 100°16.05'E, ca 610 m), 21.VIII.2011, Q.Y. Zhao leg.; 1♂ (IZCAS-Ar43092), Mohan Township, Shanggang Village, Xiaolongha, seasonal rainforest (21°24.19'N, 101°37.03'E, ca 660 m), 29.VI.2012, Q.Y. Zhao & Z.G. Chen leg.; 1♀ (IZCAS-Ar43093), same locality (21°24.33'N, 101°37.02'E, ca 800 m), 30.VI.2012, Q.Y. Zhao & Z.G. Chen leg.; 1♀ (IZCAS-Ar43094), Mengla Township, Bubang Village (21°36.64'N, 101°34.91'E, ca 820 m), 10.VII.2012, Q.Y. Zhao & Z.G. Chen leg.; 1♂ (IZCAS-Ar43095), #3 site in Mafengzhai Village (21°53.68'N, 101°17.33'E, ca 540 m), 8.V.2019, Y.F. Tong leg.; 1♀1♂ (IZCAS-Ar43096), Lüshilin Forest Park (21°53.84'N, 101°16.84'E, ca 550 m), 10.V.2019, Z.L. Bai leg.

**Figure 1.**
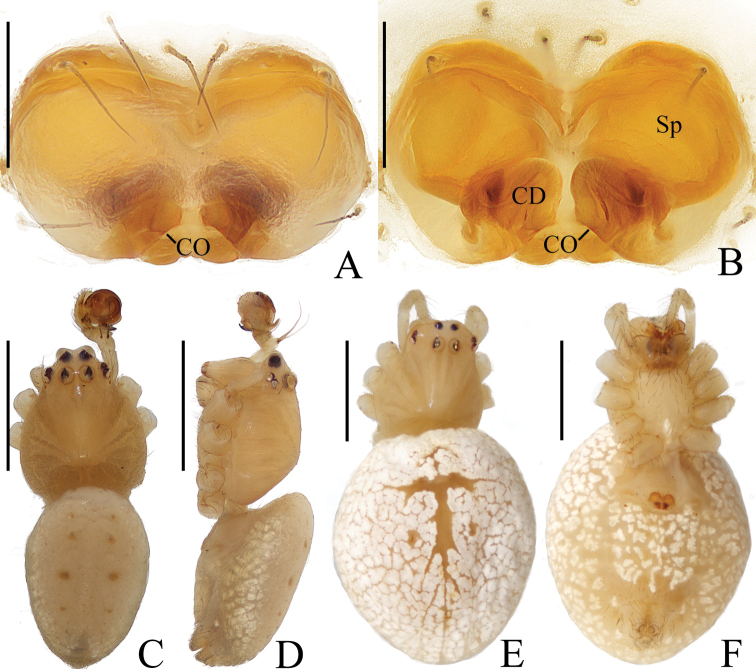
*Araneusarcuatus* sp. nov. **A, B, E, F** female paratype IZCAS-Ar43085 **C, D** male holotype **A** epigyne, ventral view **B** vulva, ventral view **C** habitus, dorsal view **D** ibid., lateral view **E** ibid., dorsal view **G** ibid., ventral view. Scale bars: 0.1 mm (**A, B**); 1 mm (**C–F**).

##### Etymology.

The specific name is derived from the Latin word “arcuatus”, meaning arcuate (curved), referring to the shape of the median apophysis in prolateral view.

##### Diagnosis.

**Figure 2.**
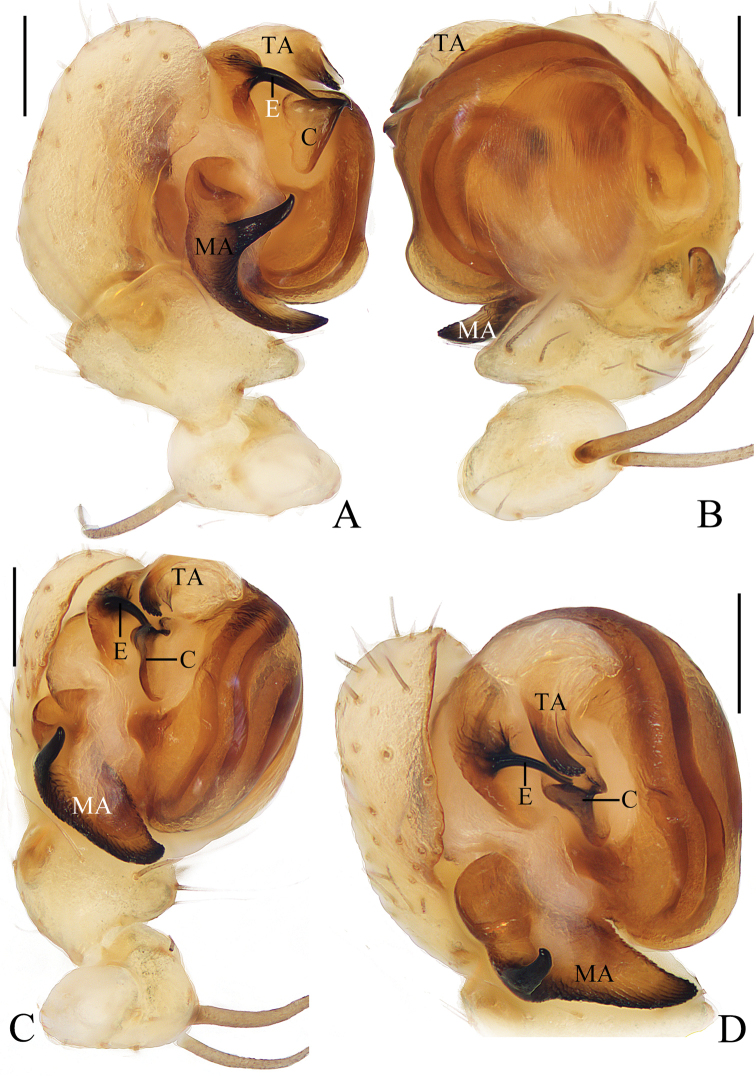
*Araneusarcuatus* sp. nov. male holotype **A** male palp, prolateral view **B** ibid., retrolateral view **C** ibid., ventral view **D** ibid., apical view. Scale bars: 0.1 mm.

**Figure 3.**
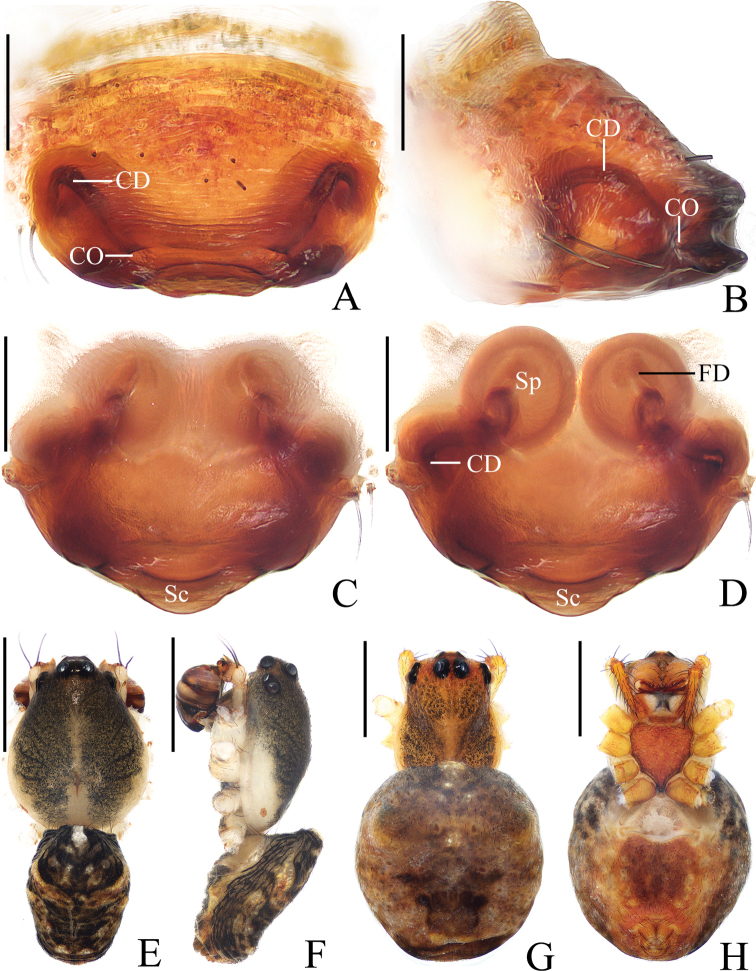
*Araneusbidentatus* sp. nov. **A–D, G, H** female paratype IZCAS-Ar43098 **E, F** male holotype **A** epigyne, ventral view **B** ibid., lateral view **C** ibid., posterior view **D** vulva, posterior view **E** habitus, dorsal view **F** ibid., lateral view **G** ibid., dorsal view **H** ibid., ventral view. Scale bars: 0.1 mm (**A–D**); 1 mm (**E–H**).

The female of the new species resembles that of *A.cucullatus* sp. nov. in appearance but can be distinguished from it as follows: 1) epigyne about 1.65 times wider than long vs about 2 times wider than long (Fig. 10A); 2) copulatory openings situated on the ventral surface vs the posterior surface (Fig. 10B); 3) spermathecae touching each other vs separated (Fig. 10C); and 4) abdomen lacking sparse, long setae vs having sparse, long setae (Fig. 10D). The male of the new species resembles that of *A.tetracanthus* sp. nov. in appearance but differs as follows: 1) median apophysis has 2 tapered tips vs 4 tapered tips (Fig. 17); 2) tegulum lacks an extension vs with a tegular extension (Fig. 17); and 3) embolus shorter than half of the bulb diameter vs longer than half of the bulb diameter (Fig. 17D).

##### Description.

**Male** (holotype, Figs 1C, D, 2, 18A–D). Total length 2.45. Carapace 1.20 long, 1.00 wide. Abdomen 1.35 long, 0.95 wide. Clypeus 0.10 high. Eye sizes and interdistances: AME 0.10, ALE 0.08, PME 0.10, PLE 0.08, AME–AME 0.13, AME–ALE 0.08, PME–PME 0.13, PME–PLE 0.10, MOA length 0.30, anterior width 0.28, posterior width 0.28. Leg measurements: I 3.85 (1.10, 1.35, 1.00, 0.40), II 3.70 (1.10, 1.30, 0.90, 0.40), III 2.45 (0.80, 0.75, 0.60, 0.30), IV 3.35 (1.05, 1.10, 0.85, 0.35). Carapace pear-shaped, yellow, cervical groove slightly distinct, fovea transverse, inner base of PMEs elevated. Chelicerae yellow, 3 promarginal and 3 retromarginal teeth. Endites almost square, yellow with very narrow, dark anterior edge, labium triangular, yellowish brown with yellow edge. Sternum cordiform, yellow with dark setae. Legs yellow without annulus, tibia I with 13 macrosetae, tibia II with 8 macrosetae. Abdomen elliptical, about 1.4 times longer than wide, in life, light yellowish green or grayish yellow when preserved with inconspicuous, white scaly spots; venter grayish yellow with inconspicuous, white scaly spots. Spinnerets yellow.

***Palp*** (Fig. 2): 2 patellar bristles; median apophysis large, arcuate in prolateral view, with two-pointed tips on opposite sides; embolus wide at base, slender and slightly curved distally; conductor triangular, concave at middle part; terminal apophysis about half the length of the bulb diameter, bifurcated distally.

**Female** (paratype IZCAS-Ar43085, Fig. 1A, B, E, F). Total length 3.40. Carapace 1.30 long, 1.15 wide. Abdomen 2.35 long, 2.05 wide. Clypeus 0.08 high. Eye sizes and interdistances: AME 0.10, ALE 0.08, PME 0.13, PLE 0.08, AME–AME 0.13, AME–ALE 0.15, PME–PME 0.15, PME–PLE 0.15, MOA length 0.33, anterior width 0.28, posterior width 0.30. Leg measurements: I 4.50 (1.30, 1.55, 1.20, 0.45), II 4.25 (1.25, 1.55, 1.05, 0.40), III 3.00 (0.95, 0.95, 0.75, 0.35), IV 4.15 (1.30, 1.40, 1.05, 0.40). Habitus similar to that of male, but white scaly spots more distinct.

***Epigyne*** (Fig. 1A, B): about 1.65 times wider than long, lacking scape; copulatory openings slit-like, close to posterior margin; copulatory ducts almost as long as the spermathecae diameter, coiled about 360°; spermathecae spherical, touching each other.

***Variation*.** Total length: ♂♂ 2.35–2.60; ♀♀ 3.15–3.40.

##### Distribution.

Known only from type localities (Yunnan, China).

#### ﻿ Araneus bidentatus sp. nov.

Taxon classificationAnimaliaAraneaeAraneidae

86324C58-1C81-544B-B479-F858B69EB31E

https://zoobank.org/516C5C7D-2580-4672-BF6C-A8B9F58F8674

Figs 3 , 4 , 18E–H

##### Type material.

***Holotype*** ♂ (IZCAS-Ar43097), China: Yunnan, Xishuangbanna, Menghai County, Menghai Township, Mengweng Village, Wengnan, secondary forest (22°4.94'N, 100°22.03'E, ca 1150 m), 2.VII.2013, Q.Y. Zhao & Z.G. Chen leg. ***Paratypes***: 1♂1♀ (IZCAS-Ar43098–43099), same data as holotype; 1♂ (IZCAS-Ar43100), Mengla County, Mohan Township, Shanggang Village, Xiaolongha, valley rainforest (21°24.25'N, 101°36.32'E, ca 760 m), 15.VI.2013, Q.Y. Zhao & Z.G. Chen leg.

##### Etymology.

The specific name is a combination of the Latin prefix “bi-” and “dentatus” (two toothed), referring to the two heavily sclerotized denticulate protuberances on the tibia of the male palp.

##### Diagnosis.

**Figure 4.**
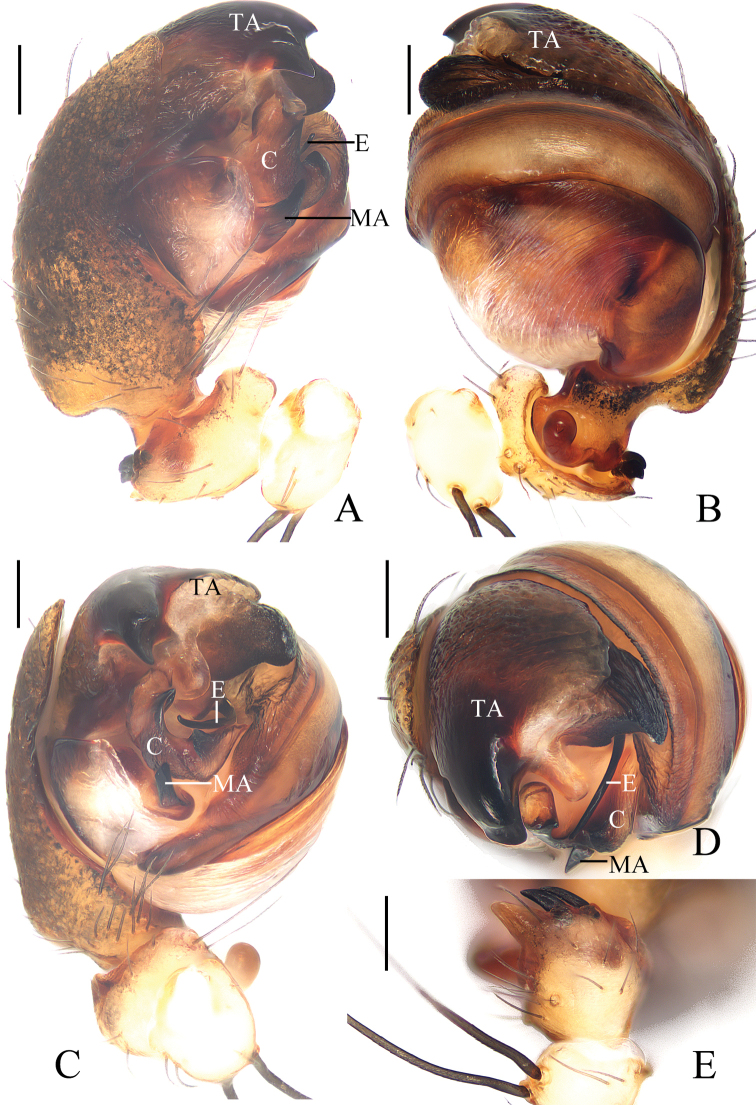
*Araneusbidentatus* sp. nov. male holotype **A** male palp, prolateral view **B** ibid., retrolateral view **C** ibid., ventral view **D** ibid., apical view **E** tibia of left male palp, retrolateral view. Scale bars: 0.1 mm.

**Figure 5.**
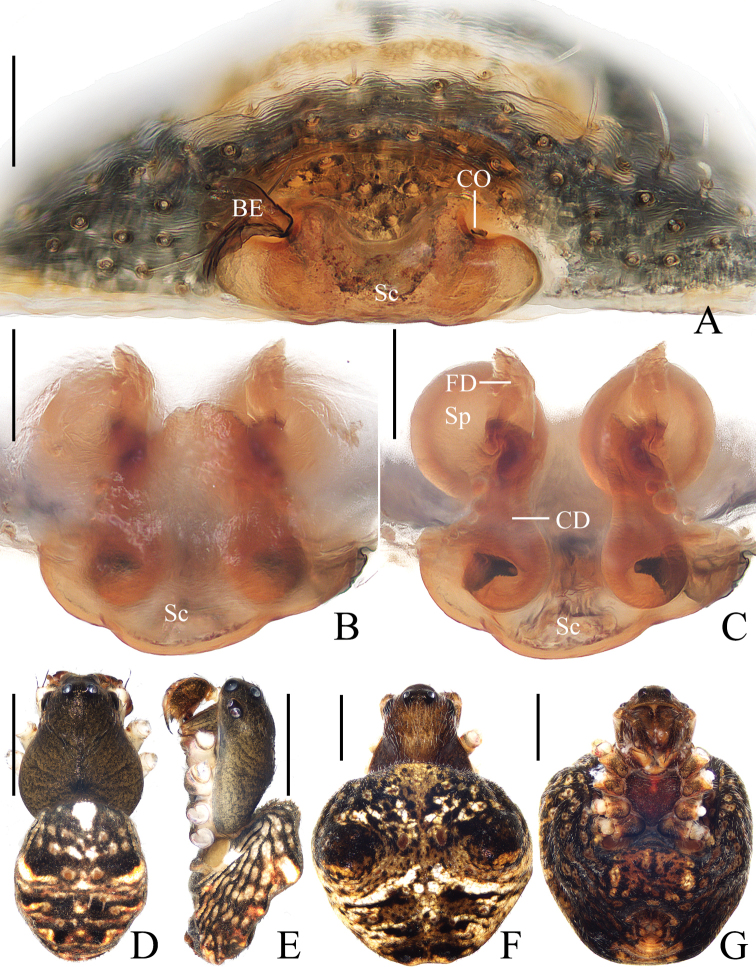
*Araneusbidentatoides* sp. nov. **A–C, F, G** female paratype IZCAS-Ar43102 **D, E** male holotype **A** epigyne, ventral view **B** ibid., posterior view **C** vulva, posterior view **D** habitus, dorsal view **E** ibid., lateral view **F** ibid., dorsal view **G** ibid., ventral view. Scale bars: 0.1 mm (**A–C**); 1 mm (**D–G**).

**Figure 6.**
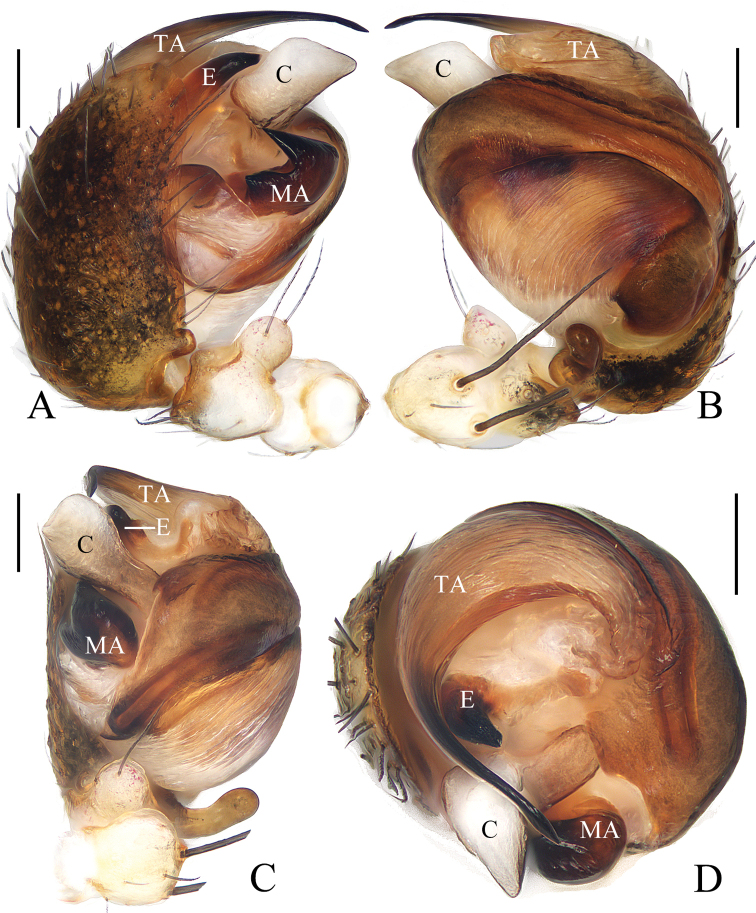
*Araneusbidentatoides* sp. nov. male holotype **A** male palp, prolateral view **B** ibid., retrolateral view **C** ibid., ventral view **D** ibid., apical view. Scale bars: 0.1 mm.

The new species resembles *A.bidentatoides* sp. nov. in appearance, but differs in the following: 1) copulatory openings located at lateral ends of the scape groove vs at anterolateral base of the scape (Fig. 5A); 2) copulatory ducts not expanded vs expanded at their origin (Fig. 5C); 3) embolus slender, a bit more slender than the patellar bristle vs embolus stout, several times bigger than the patellar bristle (Fig. 6A, C, D); 4) distal end of the terminal apophysis not tapered to a long tip vs tapered (Fig. 6); 5) median apophysis slightly curved vs curved about 90° (Fig. 6A); 6) tibia with 2 heavily sclerotized denticulate protuberances vs protuberances absent (Fig. 6A, B); and 7) fovea region and sides of thoracic region paler than thoracic region vs unicolor (Fig. 5D–G).

##### Description.

**Male** (holotype, Figs 3E, F, 4, 18E–H). Total length 2.70. Carapace 1.60 long, 1.20 wide. Abdomen 1.50 long, 0.95 wide. Clypeus 0.13 high. Eye sizes and interdistances: AME 0.13, ALE 0.10, PME 0.13, PLE 0.13, AME–AME 0.13, AME–ALE 0.10, PME–PME 0.13, PME–PLE 0.20, MOA length 0.23, anterior width 0.35, posterior width 0.35. Leg measurements: I 5.45 (1.65, 2.00, 1.15, 0.65), II 4.50 (1.45, 1.55, 0.95, 0.55), III 3.05 (1.00, 1.05, 0.55, 0.45), IV 4.15 (1.40, 1.40, 0.85, 0.50). Carapace pear-shaped, grayish black with yellow patches around fovea and on lateral edges of thoracic region, ALEs, PMEs and PLEs with black base, with pale setae, cervical groove inconspicuous. Chelicerae yellowish brown, 5 promarginal teeth, 3 retromarginal teeth. Endites and labium dark brown at base, paler distally, endites square, labium triangular. Sternum cordiform, dark brown with dark setae. Legs yellow with grayish brown annuli, tibia I with 9 macrosetae, tibia II with 11 macrosetae, tibia III with 7 macrosetae, tibia IV with 8 macrosetae. Abdomen elliptical, about 1.25 times longer than wide, covered with gray setae, dorsum yellow with white spot anteriorly and irregular black markings; venter yellow with dark brown patches. Spinnerets yellowish brown.

***Palp*** (Fig. 4): patella with 2 bristles; tibia with 2 heavily sclerotized denticulate protuberances (fig. 4B, E); paracymbium widened at base, with a finger-like tip; median apophysis shorter than the conductor, tapered to a pointed tip; embolus slender, slightly curved; conductor very wide, semicircular in prolateral view; terminal apophysis extremely large, heavily sclerotized, with 2 widely separated protuberances in apical view.

**Female** (paratype IZCAS-Ar43098, Fig. 3A–D, G, H). Total length 3.25. Carapace 1.55 long, 1.15 wide. Abdomen 2.35 long, 1.90 wide. Clypeus 0.08 high. Eye sizes and interdistances: AME 0.13, ALE 0.08, PME 0.13, PLE 0.10, AME–AME 0.13, AME–ALE 0.18, PME–PME 0.15, PME–PLE 0.25, MOA length 0.33, anterior width 0.35, posterior width 0.33. Habitus similar to that of male but with a pair of low anterolateral humps and a little paler.

***Epigyne*** (Fig. 3A–D): about 1.5 times wider than long; scape triangular, very short, about 5 times wider than long, distally with transverse groove; copulatory openings on the ends of the groove; copulatory ducts longer than the spermatheca diameter, curved; spermathecae globular, touching each other.

***Variation*.** Total length: ♂♂ 2.70–3.25.

##### Distribution.

Known only from type localities (Yunnan, China).

#### ﻿ Araneus bidentatoides sp. nov.

Taxon classificationAnimaliaAraneaeAraneidae

8636D7C8-22FC-566C-BF74-E6DEAEBB8F77

https://zoobank.org/BA43FA37-7907-40AF-AE3D-864928F7F4D3

Figs 5 , 6

##### Type material.

***Holotype*** ♂ (IZCAS-Ar43101), China: Yunnan, Xishuangbanna, Jinghong City, Mengyang Township, around Baihuashan tunnel, seasonal rainforest (22°9.51'N, 100°53.22'E, ca 890 m), 25.VI.2013, Q.Y. Zhao & Z.G. Chen leg. ***Paratype***: 1♀ (IZCAS-Ar43102), Mengla County, Menglun Township, Menglun Nature Reserve, rubber plantation (21°54.73'N, 101°16.72'E, ca 590 m), 27.V.2013, Q.Y. Zhao & Z.G. Chen leg.

##### Etymology.

The specific name is a compound of “bidentatus” and the suffix “-oides”, referring to the resemblance of this species to *A.bidentatus* sp. nov.

##### Diagnosis.

See diagnosis above for the species *A.bidentatus* sp. nov.

##### Description.

**Male** (holotype, Figs 5D, E, 6). Total length 2.75. Carapace 1.50 long, 1.15 wide. Abdomen 1.75 long, 1.25 wide. Clypeus 0.10 high. Eye sizes and interdistances: AME 0.13, ALE 0.08, PME 0.10, PLE 0.10, AME–AME 0.15, AME–ALE 0.13, PME–PME 0.23, PME–PLE 0.15, MOA length 0.35, anterior width 0.35, posterior width 0.33. Leg measurements: I 4.35 (1.35, 1.55, 0.95, 0.50), II 3.70 (1.20, 1.25, 0.75, 0.50), III 2.70 (0.90, 0.90, 0.50, 0.40), IV 3.55 (1.15, 1.20, 0.75, 0.45). Carapace pear-shaped, dark brown, with pale setae, cervical groove slightly distinct. Chelicerae dark brown, 4 promarginal teeth, 3 retromarginal teeth. Endites wider than long, reddish brown, paler distally, labium triangular, dark brown, paler distally. Sternum cordiform, dark brown with a longitudinal reddish-yellow patch, with pale setae. Legs brown with yellow annuli, tibia I with 9 macrosetae, tibia II with 10 macrosetae, tibia III with 5 macrosetae, and tibia IV with 8 macrosetae. Abdomen elliptical, about 1.4 times longer than wide, with pair of very low humps anterolaterally, covered with pale setae, dorsum yellow with large white spot anteriorly, anterior half of humps black, posterior half whitish yellow, posterior abdomen with irregular dark markings; venter yellowish brown with irregular dark markings. Spinnerets yellowish brown.

***Palp*** (Fig. 6): with 2 patellar bristles; median apophysis prominent, curved about 90° anticlockwise, distal end pointed toward tip of cymbium; embolus finger-like, tip slightly curved; conductor large, almost square in prolateral view; terminal apophysis with stout base, distally curved about 180° and tapered into tiny tip.

**Female** (paratype IZCAS-Ar43102, Fig. 5A–C, F, G). Total length 4.20. Carapace 2.20 long, 1.45 wide. Abdomen 3.40 long, 3.20 wide. Clypeus 0.13 high. Eye sizes and interdistances: AME 0.15, ALE 0.08, PME 0.13, PLE 0.10, AME–AME 0.23, AME–ALE 0.23, PME–PME 0.25, PME–PLE 0.38, MOA length 0.43, anterior width 0.45, posterior width 0.45. Leg measurements: I 5.25 (1.65, 1.90, 1.10, 0.60), II 4.65 (1.50, 1.65, 0.95, 0.55), III 3.35 (1.10, 1.15, 0.65, 0.45), IV 4.90 (1.70, 1.75, 0.95, 0.50). Habitus similar to that of male but abdomen slightly longer than wide.

***Epigyne*** (Fig. 5A–C): about 3 times wider than long; scape short, about 5 times wider than long, directed ventrally; copulatory openings concave, located at anterolateral base of scape; copulatory ducts longer than the spermatheca diameter, expanded at origin; spermathecae globular, less than half the spermatheca diameter apart.

##### Distribution.

Known only from type localities (Yunnan, China).

#### ﻿ Araneus complanatus sp. nov.

Taxon classificationAnimaliaAraneaeAraneidae

BD706A84-77BF-574D-BC66-C42291F2F1D9

https://zoobank.org/E78494DB-9CD7-41EA-980B-3B6E55064015

Figs 7 , 8 , 18I, J

##### Type material.

***Holotype*** ♂ (IZCAS-Ar43105), China: Yunnan, Xishuangbanna, Mengla County, Menglun Township, 48 km from Xishuangbanna National Nature Reserve (21°58.70'N, 101°19.75'E, ca 1090 m), 12.VIII.2011, G. Zheng leg. ***Paratype***: 1♀ (IZCAS-Ar43106), Mohan Township, Shanggang Village, Xiaolongha, secondary tropical forest (21°24.33'N, 101°37.02'E, ca 800 m), 30.VI.2012, Q.Y. Zhao & Z.G. Chen leg.

##### Etymology.

The specific name comes from the Latin word “complanatus”, meaning “flattened”, referring to the shape of the abdomen.

##### Diagnosis.

The new species resembles *A.arcuatus* sp. nov. in appearance, but can be distinguished from the latter in the following: 1) epigyne with a triangular scape vs scape absent (Fig. 1A, B); 2) copulatory openings located at the posterior surface vs ventral surface (Fig. 1A, B); 3) median apophysis with 1 tapered tip vs 2 tapered tips (Fig. 2A, C, D); and 4) 1 patellar bristle vs 2 patellar bristles (Fig. 2A–C).

##### Description.

**Figure 7.**
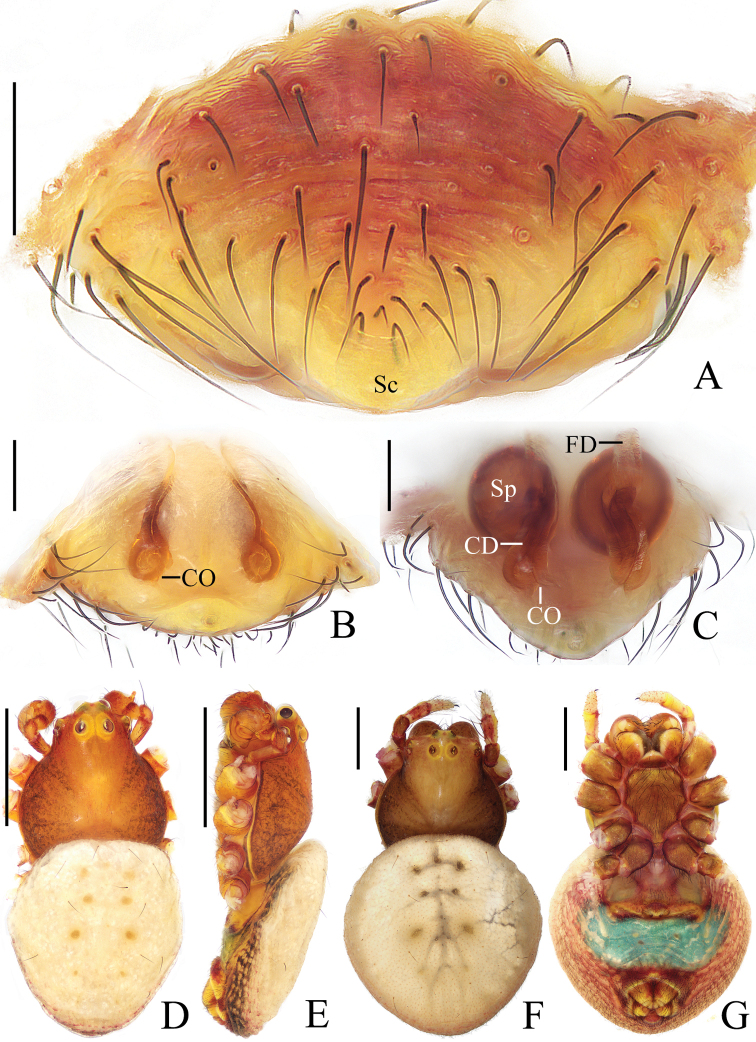
*Araneuscomplanatus* sp. nov. **A–C, F, G** female paratype IZCAS-Ar43106 **D, E** male holotype **A** epigyne, ventral view **B** ibid., posterior view **C** vulva, posterior view **D** habitus, dorsal view **E** ibid., lateral view **F** ibid., dorsal view **G** ibid., ventral view. Scale bars: 0.1 mm (**A–C**); 1 mm (**D–G**).

**Figure 8.**
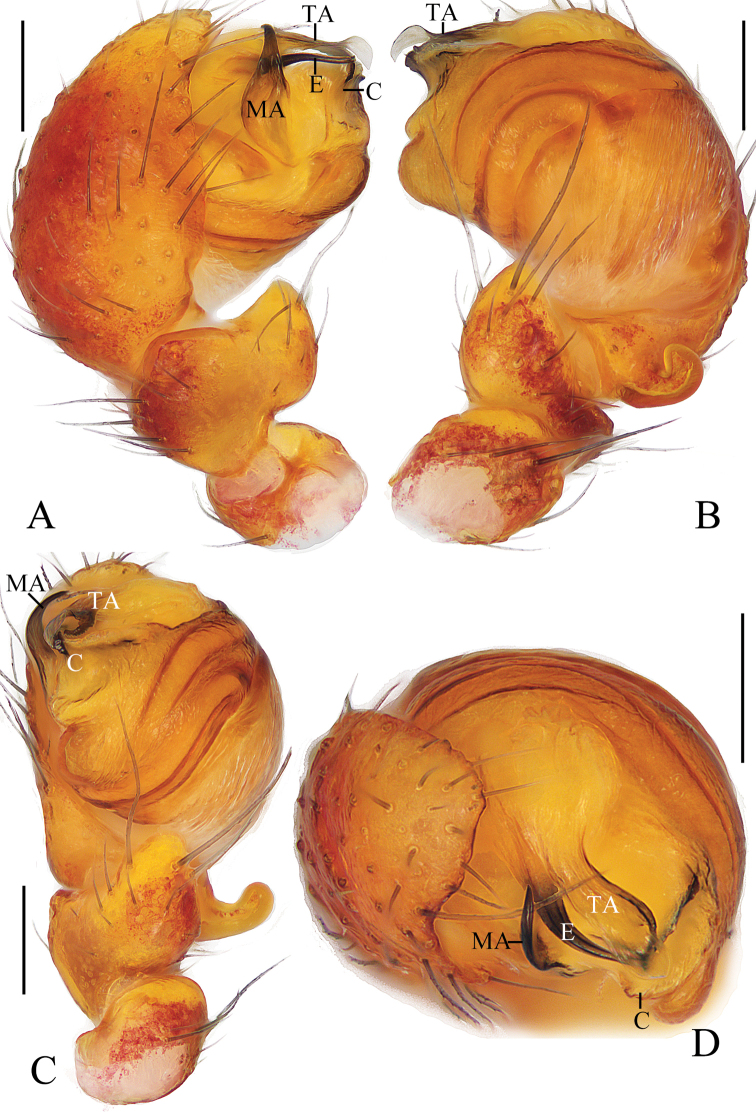
*Araneuscomplanatus* sp. nov. male holotype **A** male palp, prolateral view **B** ibid., retrolateral view **C** ibid., ventral view **D** ibid., apical view. Scale bars: 0.1 mm.

**Male** (holotype, Figs 7D, E, 8). Total length 2.75. Carapace 1.40 long, 1.25 wide. Abdomen 1.60 long, 1.45 wide. Clypeus 0.13 high. Eye sizes and interdistances: AME 0.13, ALE 0.10, PME 0.13, PLE 0.08, AME–AME 0.13, AME–ALE 0.13, PME–PME 0.15, PME–PLE 0.13, MOA length 0.33, anterior width 0.30, posterior width 0.30. Leg measurements: I 3.95 (1.15, 1.35, 0.95, 0.50), II 3.65 (1.10, 1.20, 0.90, 0.45), III 2.60 (0.80, 0.85, 0.60, 0.35), IV 3.50 (1.10, 1.15, 0.85, 0.40). Carapace pear-shaped, brown with pale patches around PMEs and dark brown patches on thoracic edges, with gray setae, cervical groove slightly distinct. Chelicerae brown, 4 promarginal and 3 retromarginal teeth. Endites and labium brown at base, paler distally, endites square, labium triangular. Sternum cordiform, yellowish brown with brown setae. Legs reddish brown, with yellowish green patches, tibia, metatarsus, and tarsus of legs I and II with a cluster of dense macrosetae. Abdomen oval, about 1.1 times longer than wide, covered with sparse, dark setae, dorsum yellow with 4 pairs of sigillae, flattened in lateral view; venter yellowish brown with light yellowish-green patch medially. Spinnerets yellow.

***Palp*** (Fig. 8): with 1 patellar bristle; median apophysis stout at base, claw like at tip; embolus slender, slightly curved; conductor broad at base, tapering to narrow tip; terminal apophysis long, membranous distally.

**Female** (paratype IZCAS-Ar43106, Figs 7A–C, F, G, 18I, J). Total length 4.95. Carapace 2.35 long, 2.15 wide. Abdomen 3.30 long, 3.30 wide. Clypeus 0.13 high. Eye sizes and interdistances: AME 0.13, ALE 0.13, PME 0.13, PLE 0.10, AME–AME 0.20, AME–ALE 0.30, PME–PME 0.23, PME–PLE 0.28, MOA length 0.45, anterior width 0.40, posterior width 0.43. Leg measurements: I 6.20 (1.85, 2.20, 1.45, 0.70), II 6.00 (1.85, 2.10, 1.40, 0.65), III 4.20 (1.40, 1.35, 0.95, 0.50), IV 6.25 (2.05, 2.10, 1.50, 0.60). Habitus similar to that of male but green patch on ventral abdomen more distinct.

***Epigyne*** (Fig. 7A–C): about 1.5 times wider than long; scape triangular, about 2.3 times wider than long; copulatory openings narrow, at posterior surface; copulatory ducts shorter than the spermatheca diameter; spermathecae globular, touching each other.

##### Distribution.

Known only from type localities (Yunnan, China).

#### ﻿ Araneus corrugis sp. nov.

Taxon classificationAnimaliaAraneaeAraneidae

0D5B4551-3BF4-55E6-9AB1-8E8A04C025DD

https://zoobank.org/F66E9921-63C5-4F23-81B9-692C261258CC

Fig. 9

##### Type material.

***Holotype*** ♀ (IZCAS-Ar43107), China: Yunnan, Xishuangbanna, Mengla County, Menglun Township, Menglun Nature Reserve, Lüshilin Forest Park, limestone tropical seasonal rainforest (21°54.68'N, 101°16.95'E, ca 640 m), 10.VIII.2018 night, C. Wang leg. ***Paratype***: 1♀ (IZCAS-Ar43108), XTBG, eastern part (21°54.07'N, 101°16.36'E, ca 540 m), 22.VII.2018, X.Q. Mi leg.

##### Etymology.

The specific name comes from the Latin word “corrugis”, meaning “wrinkled”, referring to the texture of the epigyne basally; adjective.

##### Diagnosis.

**Figure 9.**
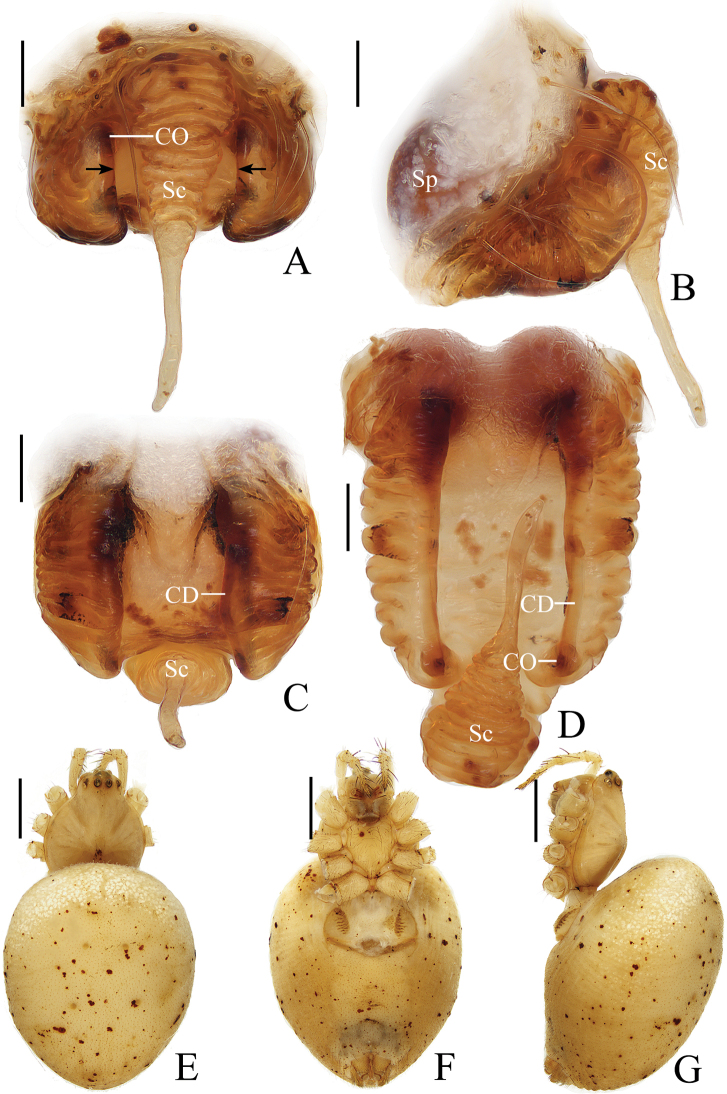
*Araneuscorrugis* sp. nov. female holotype **A** epigyne, ventral view **B** ibid., lateral view **C** ibid., posterior view **D** vulva, posterior view **E** habitus, dorsal view **F** ibid., ventral view **G** ibid., lateral view. Scale bars: 0.1 mm (**A–D**); 1 mm (**E–G**).

The new species resembles *A.gratiolus* Yin, Wang, Xie & Peng, 1990 in appearance, but it can be distinguished from the latter by the following: 1) distal part of the scape finger-like vs triangular (Yin et al. 1990: figs 79, 80); 2) distal part of the scape not grooved vs grooved (Yin et al. 1990: figs 79, 80); and 3) inner edge of the lateral plate straight in ventral view (arrows in Fig. 9A) vs arcuate (Yin et al. 1990: fig. 79).

##### Description.

**Female** (holotype, Fig. 9). Total length 5.20. Carapace 2.00 long, 1.70 wide. Abdomen 3.80 long, 3.20 wide. Clypeus 0.13 high. Eye sizes and interdistances: AME 0.18, ALE 0.13, PME 0.15, PLE 0.13, AME–AME 0.13, AME–ALE 0.13, PME–PME 0.10, PME–PLE 0.18, MOA length 0.38, anterior width 0.38, posterior width 0.35. Leg measurements: I 11.20 (3.00, 3.50, 3.60, 1.10), II 9.30 (2.70, 3.00, 2.70, 0.90), III 5.40 (1.70, 1.80, 1.30, 0.60), IV 9.10 (2.90, 3.00, 2.40, 0.80). Carapace pear-shaped, yellow, with pale setae, cervical groove distinct. Chelicerae yellowish brown, 4 promarginal and 3 retromarginal teeth. Endites almost rectangular, yellowish brown at base and paler distally, labium triangular, grayish brown, paler distally. Sternum cordiform, yellow, with dark setae. Legs yellow without annulus. Abdomen oval, rounded anteriorly, slightly pointed posteriorly, with 4 pairs of sigillae, yellow with white scaly spots anteriorly; venter yellow. Spinnerets yellow.

***Epigyne*** (Fig. 9A–D): scape exceeding beyond the epigastric furrow, broad and wrinkled at base, distal half finger-like; copulatory openings narrow, near lateral edges of scape base; copulatory ducts 2 times longer than the spermatheca diameter; spermathecae globular, touching each other.

***Variation*.** Total length: ♀♀ 5.20–6.70.

##### Distribution.

Known only from type localities (Yunnan, China).

#### ﻿ Araneus cucullatus sp. nov.

Taxon classificationAnimaliaAraneaeAraneidae

937655B0-12CA-5807-B600-8567E17D3795

https://zoobank.org/0B06353E-D025-4742-BA39-D1CAAD167DFB

Figs 10 , 18K, L

##### Type material.

***Holotype*** ♀ (IZCAS-Ar43109), China: Yunnan, Xishuangbanna, Mengla County, Menglun Township, Menglun Nature Reserve, Lüshilin Forest Park, limestone seasonal rainforest (21°54.56'N, 101°16.86'E, ca 610 m), 29.XI.2009, G. Tang leg. ***Paratype***: 1♀ (IZCAS-Ar43110), Lüshilin Forest Park (21°54.71'N, 101°16.90'E, ca 660 m), 13.XI.2009, G. Tang leg.

##### Etymology.

The specific name comes from the Latin word “cucullatus”, meaning “hooded”, referring to the hood on the ventral surface of the epigyne.

##### Diagnosis.

The new species resembles *A.arcuatus* sp. nov. in appearance, but it can be distinguished from the latter by the following: 1) epigyne about 2 times wider than long vs about 1.65 times wider than long (Fig. 1A, B); 2) copulatory openings situated on posterior surface vs ventral surface (Fig. 1A, B); 3) spermathecae separated vs touching each other (Fig. 1B); and 4) abdomen with sparse, long setae vs lacking sparse, long setae (Fig. 1E).

##### Description.

**Figure 10.**
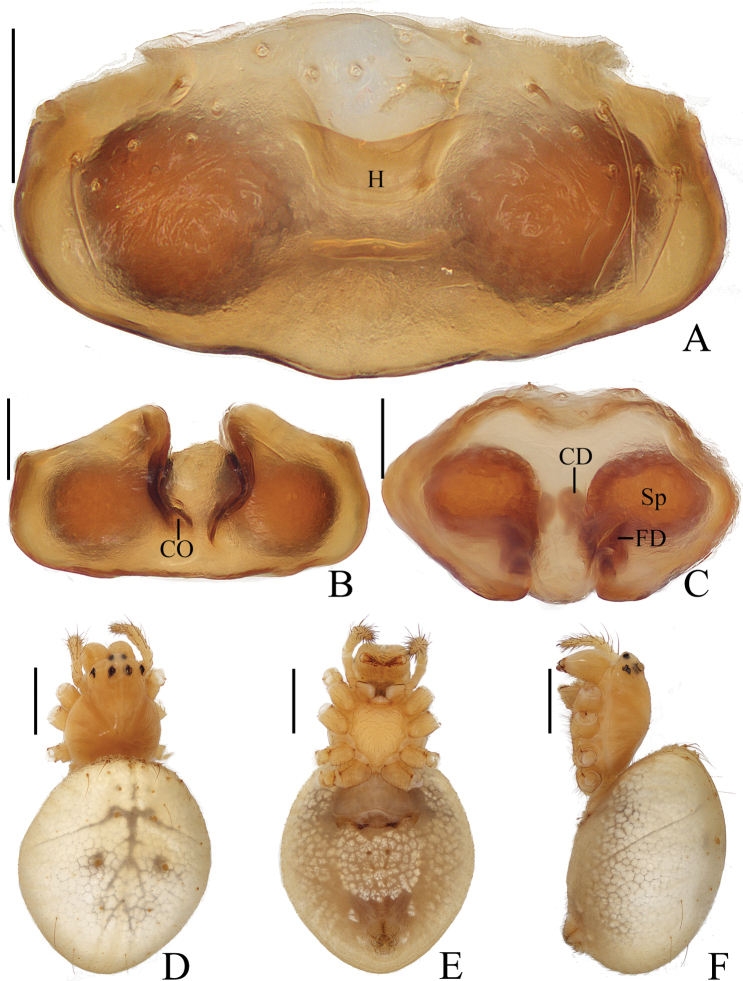
*Araneuscucullatus* sp. nov. female holotype **A** epigyne, ventral view **B** ibid., posterior view **C** vulva, dorsal view **D** habitus, dorsal view **E** ibid., ventral view **F** ibid., lateral view. Scale bars: 0.1 mm (**A–C**); 1 mm (**D–F**).

**Female** (holotype, Fig. 10, paratype Ar43110, Fig. 18K, L). Total length 4.80. Carapace 1.90 long, 1.50 wide. Abdomen 3.30 long, 2.90 wide. Clypeus 0.13 high. Eye sizes and interdistances: AME 0.13, ALE 0.10, PME 0.13, PLE 0.10, AME–AME 0.15, AME–ALE 0.23, PME–PME 0.15, PME–PLE 0.20, MOA length 0.38, anterior width 0.33, posterior width 0.38. Leg measurements: I 5.10 (1.40, 1.80, 1.30, 0.60), II 4.90 (1.40, 1.70, 1.20, 0.60), III 3.60 (1.10, 1.10, 0.90, 0.50), IV 4.80 (1.40, 1.70, 1.20, 0.50). Carapace pear-shaped, yellow, cervical groove distinct. Chelicerae yellow, 4 promarginal teeth, 3 retromarginal teeth. Endites almost rectangular, yellow with dark anterior edge, labium triangular, yellow. Sternum cordiform, yellow, with dark setae. Legs yellow without annulus, distal tibia, metatarsus, and tarsus of legs I and II with a cluster of dense macrosetae. Abdomen elliptical, whitish yellow, with sparse dark setae; venter grayish yellow with white scaly spots. Spinnerets grayish yellow.

***Epigyne*** (Fig. 10A–C): about 2 times wider than long, with a hood; copulatory openings concave, at middle part of posterior surface; copulatory ducts slender, twisted at origin; spermathecae nearly globular, half the diameter apart.

***Variation*.** Total length: ♀♀ 4.30–4.80.

##### Distribution.

Known only from the type locality (Yunnan, China).

#### ﻿ Araneus minisculus sp. nov.

Taxon classificationAnimaliaAraneaeAraneidae

B9C3897F-81DF-5125-9030-E26B7A60BC51

https://zoobank.org/3D55612E-E294-42A2-80F0-8CA8110CA840

Figs 11 , 19A, B

##### Type material.

***Holotype*** ♂ (IZCAS-Ar43112), China: Yunnan, Xishuangbanna, Mengla County, Mohan Township, Shanggang Village, Xiaolongha, seasonal rainforest (21°24.19'N, 101°36.26'E, ca 720 m), 4.VII.2012, Q.Y. Zhao & Z.G. Chen leg. ***Paratypes***: 1♂ (IZCAS-Ar43113), Menghai County, Menghai Township, Manzhen Village, Mandazhai, secondary forest (22°1.70'N, 100°23.70'E, ca 1190 m), 28.VII.2012, Q.Y. Zhao & Z.G. Chen leg.; 1♂ (IZCAS-Ar43114), Mengla County, Menglun Township, Menglun Nature Reserve, secondary tropical forest, *Anogeissusacuminate* plantation (about 20 years old), G213 roadside (21°54.03'N, 101°16.89’E, ca 610 m), 2.VIII.2018, Z.L. Bai leg.

##### Etymology.

The specific name is from the Latin word “minisculus”, meaning “small”, referring to the relatively small total length of the habitus.

##### Diagnosis.

The new species can be distinguished from any other congeners by the following combination of characters: 1) male palp with a cluster of tibial bristles; 2) dorsal abdomen with 2 pairs of longitudinal, narrow, pale patches; and 3) small total body length.

##### Description.

**Figure 11.**
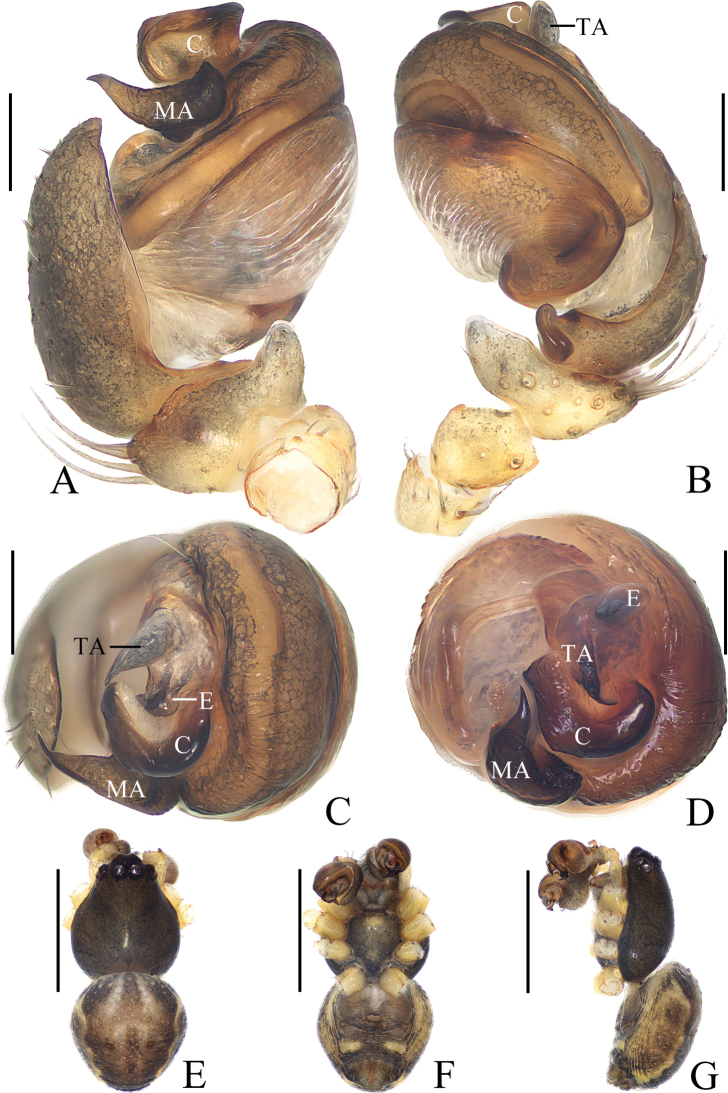
*Araneusminisculus* sp. nov. holotype **A** male palp, prolateral view **B** ibid., retrolateral view **C** ibid., apical view **D** ibid., expanded, apical view **E** habitus, dorsal view **F** ibid., ventral view **G** ibid., lateral view. Scale bars: 0.1 mm (**A–D**); 1 mm (**E–G**).

**Male** (holotype, Figs 11, 19A, B). Total length 1.90. Carapace 1.05 long, 0.85 wide. Abdomen 1.05 long, 0.90 wide. Clypeus 0.05 high. Eye sizes and interdistances: AME 0.10, ALE 0.05, PME 0.08, PLE 0.05, AME–AME 0.10, AME–ALE 0.13, PME–PME 0.05, PME–PLE 0.05, MOA length 0.25, anterior width 0.23, posterior width 0.20. Leg measurements: I 3.50 (1.05, 1.20, 0.80, 0.45), II 2.90 (0.85, 1.00, 0.65, 0.40), III 1.70 (0.55, 0.55, 0.30, 0.30), IV 2.35 (0.75, 0.75, 0.50, 0.35). Carapace pear-shaped, dark brown, with pale setae, cervical groove inconspicuous. Chelicerae grayish brown, 4 promarginal teeth, 3 retromarginal teeth. Endites and labium grayish brown at base, paler distally, endites square, labium triangular. Sternum cordiform, dark brown with yellow patch anteriorly, with pale setae. Legs yellow to yellowish brown, without annulus. Abdomen broadly oval, covered with pale setae, dorsum grayish brown with two pairs of longitudinal, narrow, pale patches; venter grayish brown with a pair of yellow patches. Spinnerets grayish brown.

***Palp*** (Fig. 11A–D): with 1 patellar bristle and a cluster of tibial bristles; median apophysis transverse, with long, tapered spur; embolus short, broad at base; conductor large, heavily sclerotized, curved; terminal apophysis small, about equal length to embolus.

***Variation*.** Total length: ♂♂ 1.80–1.90.

##### Distribution.

Known only from type localities (Yunnan, China).

#### ﻿ Araneus ovoideus sp. nov.

Taxon classificationAnimaliaAraneaeAraneidae

9C5A7FA9-8308-55AF-9A73-9805C94E7D37

https://zoobank.org/DB876BBB-1F7B-4467-9CAB-FCA7ED57318B

Figs 12 , 19C, D

##### Type material.

***Holotype*** ♀ (IZCAS-Ar43115), China: Yunnan, Xishuangbanna, Mengla County, Menglun Township, Menglun Nature Reserve, low evergreen forest along G213 roadside (21°53.79'N, 101°17.15'E, ca 590 m), 27.XI.2009, G. Tang leg. ***Paratypes***: 1♀ (IZCAS-Ar43116), Lüshilin, secondary tropical seasonal moist forest (21°54.39'N, 101°16.72'E, ca 690 m), 6.VIII.2011, G. Zheng leg.; 1♀ (IZCAS-Ar43117), secondary tropical forest around garbage dump (21°54.17'N, 101°16.87'E, ca 610 m), 31.VII.2018, Z.L. Bai leg.; 1♀ (IZCAS-Ar43118), XTBG, near the benchland (21°54.24'N, 101°15.99'E, ca 540 m), 13.V.2019, Z.L. Bai leg.

##### Etymology.

The specific name is derived from the Latin word “ovoideus”, meaning “oval”, referring to the shape of the spermathecae.

##### Diagnosis.

The new species can be distinguished from its congeners by the following combination of characters: 1) epigyne triangular; 2) a very large pair of spermathecae; 3) posterior abdomen with 1 yellow transverse patch and 2 yellow longitudinal patches; and 4) ventral abdomen with a square white patch.

##### Description.

**Figure 12.**
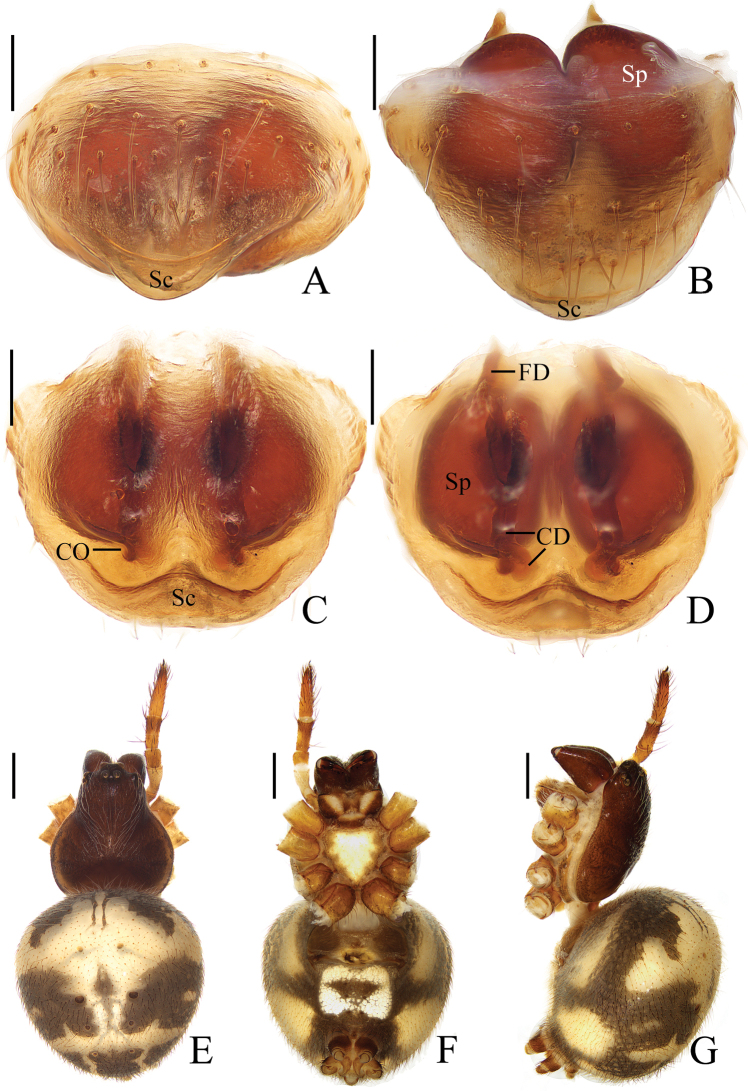
*Araneusovoideus* sp. nov. female holotype **A** epigyne, ventral view **B** ibid., anterior view **C** ibid., posterior view **D** vulva, posterior view **E** habitus, dorsal view **F** ibid., ventral view **G** ibid., lateral view. Scale bars: 0.1 mm (**A–D**); 1 mm (**E–G**).

**Female** (holotype, Figs 12, 19C, D). Total length 6.80. Carapace 3.10 long, 2.70 wide. Abdomen 4.00 long, 4.10 wide. Clypeus 0.10 high. Eye sizes and interdistances: AME 0.20, ALE 0.15, PME 0.18, PLE 0.15, AME–AME 0.18, AME–ALE 0.20, PME–PME 0.08, PME–PLE 0.35, MOA length 0.53, anterior width 0.53, posterior width 0.40. Leg measurements: I 10.60 (3.10, 3.90, 2.60, 1.00), II 9.90 (3.00, 3.50, 2.50, 0.90), III 6.10 (2.00, 2.10, 1.30, 0.70), IV 9.10 (3.00, 3.10, 2.20, 0.80). Carapace pear-shaped, dark brown, with dense, pale setae. Chelicerae dark brown, 4 promarginal teeth, 3 retromarginal teeth. Endites and labium dark brown basally, yellow distally, endites almost square, labium triangular. Sternum cordiform, yellow with yellowish-brown margins. Legs yellow with wide, brown annuli. Abdomen broadly oval, with dark setae, dorsum whitish yellow, with 2 pairs of grayish-brown patches anteriorly and medially, posteriorly with three grayish-brown patches; venter grayish brown, square white patch medially. Spinnerets brown.

***Epigyne*** (Fig. 12A–D): triangular, about 1.5 times wider than long, with very short, rimmed scape; copulatory openings concave, at posterior surface; copulatory ducts twisted at origin; spermathecae oval, touching each other.

***Variation*.** Total length: ♀♀ 6.80–7.70.

##### Distribution.

Known only from type localities (Yunnan, China).

#### ﻿ Araneus pseudodigitatus sp. nov.

Taxon classificationAnimaliaAraneaeAraneidae

483EA790-B1FA-56B2-8D55-47CF64C44D68

https://zoobank.org/65E73381-15A3-4C6B-AE4F-CDF75A6B01D0

**Figure 13.**
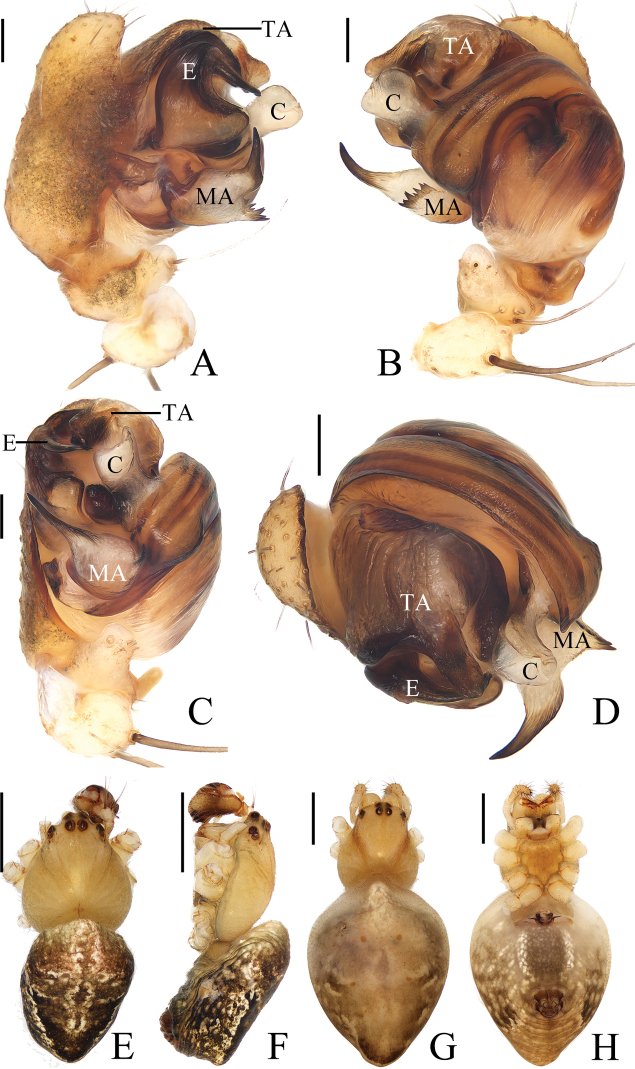
*Araneuspseudodigitatus* sp. nov. **A–F** male holotype **G, H** female paratype IZCAS-Ar43125 **A** male palp, prolateral view **B** ibid., retrolateral view **C** ibid., ventral view **D** ibid., apical view **E** habitus, dorsal view **F** ibid., lateral view **G** ibid., dorsal view **H** ibid., ventral view. Scale bars: 0.1 mm (**A–D**); 1 mm (**E–H**).

**Figure 14.**
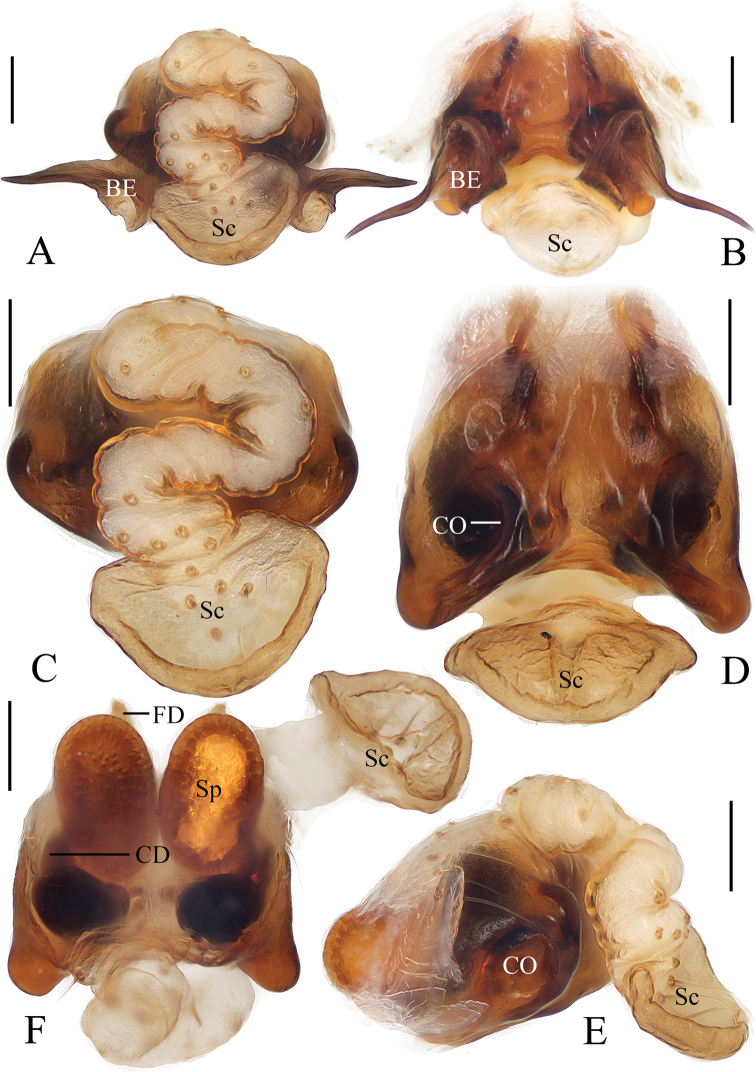
*Araneuspseudodigitatus* sp. nov. **A, C–F** female paratype IZCAS-Ar43125 **B** female paratype IZCAS-Ar43126 **A** epigyne, ventral view **B** ibid., posterior view **C** ibid., ventral view **D** ibid., posterior view **E** ibid., lateral view **F** vulva, anterior view. Scale bars: 0.1 mm.

Figs 13 , 14 , 19E–H

##### Type material.

***Holotype*** ♂ (IZCAS-Ar43119), China: Yunnan, Xishuangbanna, Mengla County, Menglun Township, Menglun Nature Reserve, XTBG, #6 site around the dump (21°54.33'N, 101°16.79'E, ca 620 m), 7.V.2019, Y.F. Tong leg. ***Paratypes***: 1♂1♀ (IZCAS-Ar43120-Ar43121), Masuoxing Village (21°54.02'N, 101°16.90'E, ca 560 m), 27.IV.2019, Y.F. Tong leg.; 1♀ (IZCAS-Ar43122), #4 site around the dump (21°54.34'N, 101°16.79'E, ca 620 m), 2.V.2019, Y.F. Tong leg.; 1♂1♀ (IZCAS-Ar43123-Ar43124), #2 site in Mafengzhai Village (21°55.83'N, 101°14.93'E, ca 540 m), 4.V.2019, Y.F. Tong leg.; 2 ♀ (IZCAS-Ar43125-Ar43126) Lüshilin Forest Park (21°53.84'N, 101°16.84'E, ca 550 m), 10.V.2019, Z.L. Bai leg.

##### Etymology.

The specific name is a combination of the Latin words “pseudo” and “digitatus”, referring to the resemblance of this species with *A.digitatus* Liu, Irfan, Yang & Peng, 2019; adjective.

##### Diagnosis.

The new species resembles *A.digitatus* in appearance, but it differs by the following: 1) copulatory openings on the posterior surface vs on the ventral surface (Liu et al. 2019: fig. 8A, B); 2) embolus curved clockwise about 135° in prolateral view vs curved <90° (Liu et al. 2019: fig. 7A, C); and 3) abdomen posteriorly pointed in both sexes vs blunt (Liu et al. 2019: fig. 6A, C).

##### Description.

**Male** (holotype, Figs 13A–F, 19E–H). Total length 3.15. Carapace 1.70 long, 1.40 wide. Abdomen 1.75 long, 1.40 wide. Clypeus 0.13 high. Eye sizes and interdistances: AME 0.13, ALE 0.10, PME 0.13, PLE 0.10, AME–AME 0.13, AME–ALE 0.13, PME–PME 0.10, PME–PLE 0.18, MOA length 0.33, anterior width 0.35, posterior width 0.28. Leg measurements: I 5.50 (1.75, 2.05, 1.15, 0.55), II 5.10 (1.60, 1.85, 1.10, 0.55), III 3.05 (1.05, 1.00, 0.60, 0.40), IV 4.35 (1.45, 1.45, 1.00, 0.45). Carapace pear-shaped, yellow with brown eye bases, with pale setae, cervical groove slightly distinct, fovea longitudinal. Chelicerae yellow, 4 promarginal teeth, 3 retromarginal teeth. Endites yellow, labium yellowish brown, paler distally. Sternum cordiform, yellow. Legs yellow to brown without annulus, tibia I with 10 macrosetae, tibia II with 8 macrosetae, tibia III with 8 macrosetae, tibia IV with 9 macrosetae. Abdomen somewhat long, triangular, about 1.25 times longer than wide, with pale setae, dorsum with a longitudinal grayish-yellow patch anteriorly, and a triangular grayish-brown patch posteriorly; venter grayish brown, with a pair of whitish-yellow longitudinal patches, laterally yellow with irregular gray patches. Spinnerets grayish brown.

***Palp*** (Fig. 13A–D): with 2 patellar bristles; median apophysis large, bifurcated, 1 long, 1 short with serrated tip; embolus large, curved about 135° at middle, tapered distally; conductor somewhat rectangular, with a spur at base; terminal apophysis extremely large, about as long as the bulb diameter.

**Female** (paratype IZCAS-Ar43125, Figs 13G, H, 14A, C–F, paratype IZCAS-Ar43125, Fig. 14B). Total length 5.10. Carapace 2.00 long, 1.50 wide. Abdomen 3.60 long, 2.70 wide. Clypeus 0.13 high. Eye sizes and interdistances: AME 0.13, ALE 0.10, PME 0.13, PLE 0.10, AME–AME 0.13, AME–ALE 0.25, PME–PME 0.10, PME–PLE 0.30, MOA length 0.33, anterior width 0.33, posterior width 0.30. Leg measurements: I 5.50 (1.70, 2.00, 1.20, 0.60), II 5.10 (1.60, 1.90, 1.00, 0.60), III 3.30 (1.10, 1.10, 0.70, 0.40), IV 4.90 (1.50, 1.80, 1.10, 0.50). Habitus similar to that of male but a little pale.

***Epigyne*** (Fig. 14): with long, tortuous scape, spoon-shaped, rimmed at tip; copulatory openings deeply concave, at posterior surface; copulatory ducts curved, extremely expanded at origin; spermathecae kidney-shaped, touching each other.

***Variation*.** Total length: ♂♂ 3.10–3.40; ♀♀ 3.90–6.10.

##### Distribution.

known only from type localities (Yunnan, China).

#### ﻿ Araneus semiorbiculatus sp. nov.

Taxon classificationAnimaliaAraneaeAraneidae

A471CA67-056A-5D5A-ADC7-D5B48BE12F89

https://zoobank.org/AF3DF4B2-B1E8-4451-90D6-EC3F8044B4FB

Figs 15 , 19I, J

##### Type material.

***Holotype*** ♀ (IZCAS-Ar43127), China: Yunnan, Xishuangbanna, Mengla County, Menglun Township, Menglun Nature Reserve, XTBG, 26.XII.2006, S.Q. Li leg. ***Paratypes***: 1♀ (IZCAS-Ar43128), same data as holotype; 1♀ (IZCAS-Ar43129), Menglun Township, 55 km from Xishuangbanna National Nature Reserve, valley rainforest (21°57.68'N, 101°12.03'E, ca 720 m), 12.VI.2013, Q.Y. Zhao & Z.G. Chen leg.

##### Etymology.

The specific name is from the Latin word “semiorbiculatus”, meaning “semicircle”, referring to the shape of the scape.

##### Diagnosis.

The new species resembles *A.bidentatus* sp. nov., but it can be distinguished from the latter by the following: 1) copulatory openings situated lateral to posterior surface vs at lateral end of the scape groove (Fig. 3A, B); 2) distal end of the scape not grooved vs grooved (Fig. 3A, B); and 3) scape width equal to 2 diameters of a spermatheca vs width about 1.3 times of a spermatheca diameter (Fig. 3D).

##### Description.

**Figure 15.**
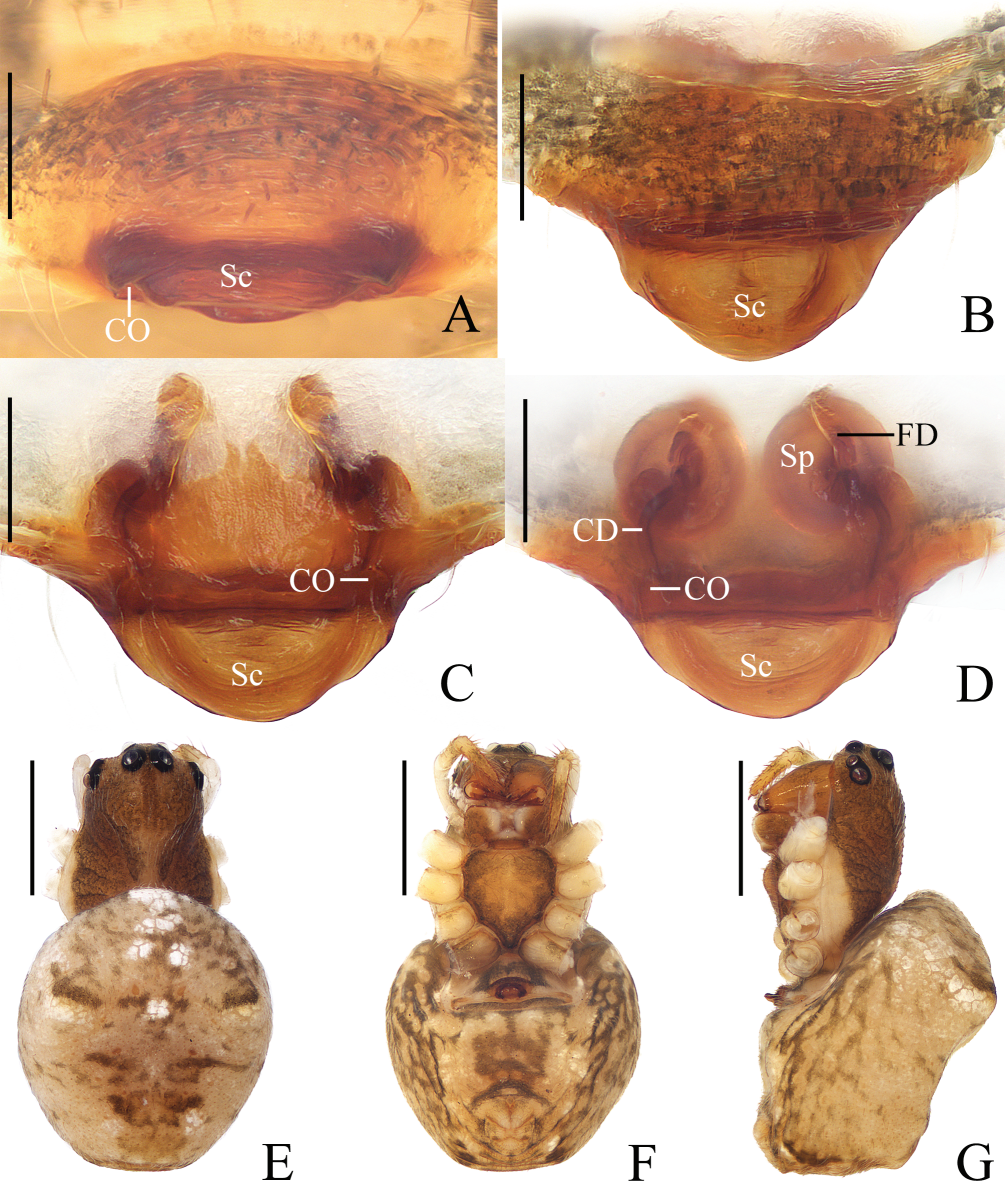
*Araneussemiorbiculatus* sp. nov. female holotype **A** epigyne, ventral view **B** ibid., anterior view **C** ibid., posterior view **D** vulva, posterior view **E** habitus, dorsal view **F** ibid., ventral view **G** ibid., lateral view. Scale bars: 0.1 mm (**A–D**); 1 mm (**E–G**).

**Female** (holotype, Figs 15, 19I, J). Total length 3.25. Carapace 1.75 long, 1.30 wide. Abdomen 2.40 long, 1.90 wide. Clypeus 0.05 high. Eye sizes and interdistances: AME 0.13, ALE 0.10, PME 0.13, PLE 0.13, AME–AME 0.13, AME–ALE 0.18, PME–PME 0.13, PME–PLE 0.25, MOA length 0.33, anterior width 0.35, posterior width 0.35. Leg measurements: I 4.80 (1.45, 1.70, 1.00, 0.65), II 4.15 (1.25, 1.45, 0.85, 0.60), III 3.00 (0.95, 1.00, 0.55, 0.50), IV 4.20 (1.40, 1.40, 0.85, 0.55). Carapace pear-shaped, brown with yellow patches around the fovea and laterally on thoracic region, ALEs, PMEs, and PLEs with black base, with pale setae, cervical groove distinct. Chelicerae yellowish brown, 5 promarginal teeth, 3 retromarginal teeth. Endites and labium brown at base, paler distally, endites square, labium triangular. Sternum cordiform, yellow with dark brown edges, with dark setae. Legs yellow with inconspicuous grayish-brown annuli. Abdomen elliptical, about 1.25 times longer than wide, covered with gray setae, dorsum grayish yellow with large white spot anteriorly and irregular black markings; venter yellow with dark brown patches. Spinnerets yellow.

***Epigyne*** (Fig. 15A–D): about 1.8 times wider than long, with a semicircular scape, round copulatory openings at lateral to posterior surface; copulatory duct length about equal to the spermatheca diameter, slightly curved; spermathecae globular, less than half the spermathecae diameter apart.

***Variation*.** Total length: ♀♀ 3.20–3.25.

##### Distribution.

Known only from type localities (Yunnan, China).

#### ﻿ Araneus tetracanthus sp. nov.

Taxon classificationAnimaliaAraneaeAraneidae

2E1718CE-ACA4-5BFC-B9CB-8CDC65C17CF4

https://zoobank.org/71CE9BA2-64AC-48B3-892D-C70A9DEF0590

**Figure 16.**
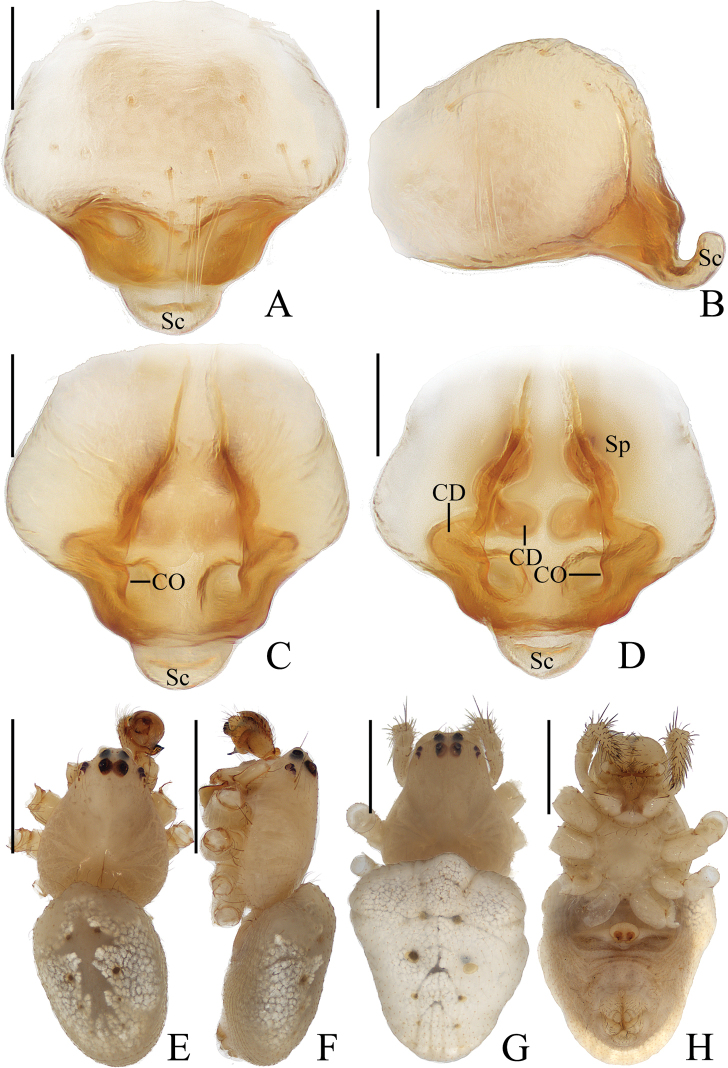
*Araneustetracanthus* sp. nov. **A–D, G, H** female paratype IZCAS-Ar43135 **E, F** male holotype **A** epigyne, ventral view **B** ibid., lateral view **C** ibid., posterior view **D** vulva, posterior view **E** habitus, dorsal view **F** ibid., lateral view **G** ibid., dorsal view **H** ibid., ventral view. Scale bars: 0.1 mm (**A–D**); 1 mm (**E–H**).

**Figure 17.**
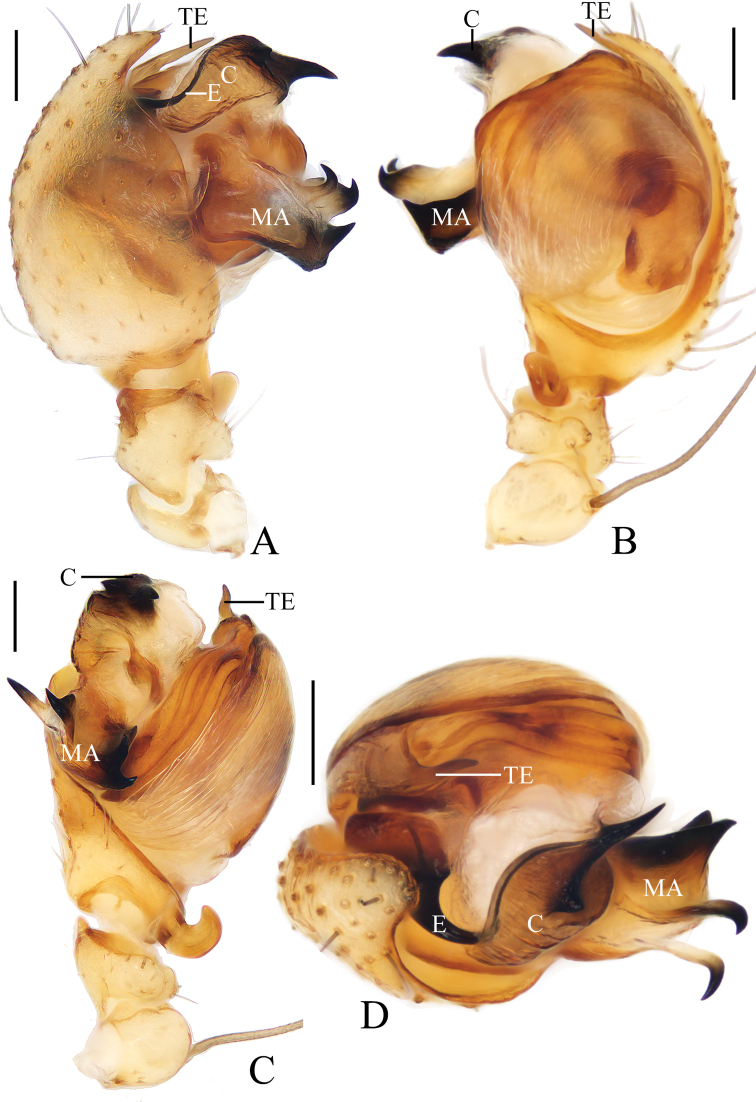
*Araneustetracanthus* sp. nov. male holotype **A** male palp, prolateral view **B** ibid., retrolateral view **C** ibid., ventral view **D** ibid., apical view. Scale bars: 0.1 mm.

Figs 16 , 17 , 19K–N

##### Type material.

***Holotype*** ♂ (IZCAS-Ar43130), China: Yunnan, Xishuangbanna, Mengla County, Menglun Township, Menglun Nature Reserve, Lüshilin Forest Park (21°53.84'N, 101°16.84'E, ca 550 m), 10.V.2019, Z.L. Bai leg. ***Paratypes***: 1♂ (IZCAS-Ar43131), G213 roadside (21°54.39'N, 101°16.80'E, ca 630 m), 22.XI.2009, G. Tang leg.; 1♂ (IZCAS-Ar43131), G213 roadside (21°53.67'N, 101°16.98'E, ca 590 m), 26.XI.2009, G. Tang leg.; 1♂ (IZCAS-Ar43133), G213 roadside (21°54.09'N, 101°17.02'E, ca 570 m), 28.XI.2009, G. Tang leg.; 1♂ (IZCAS-Ar43134), G213 roadside, bamboo plantation (21°53.64'N, 101°16.94'E, ca 580 m), 3.XII.2009, G. Tang leg.; 1♂1♀ (IZCAS-Ar43135–43136), Masuoxing Village (21°54.02'N, 101°16.90'E, ca 560 m), 27.IV.2019, Y.F. Tong leg.; 1♂ (IZCAS-Ar43137), #1 site in Mafengzhai Village (21°53.44'N, 101°17.40'E, ca 540 m), 29.IV.2019, Y.F. Tong leg.; 1♂ (IZCAS-Ar43138), Lüshilin, secondary tropical seasonal moist forest (21°54.39'N, 101°16.72'E, ca 690 m), 6.VIII.2011, G. Zheng leg.; 1♂ (IZCAS-Ar43139), Mohan Township, Shanggang Village, Xiaolongha, seasonal rainforest (21°24.19'N, 101°37.03'E, ca 660 m), 29.VI.2012, Q.Y. Zhao & Z.G. Chen leg.

##### Etymology.

The specific name comes from the Latin word “tetracanthus”, meaning “four spines”, referring to the four protuberances on the median apophysis.

##### Diagnosis.

The new species resembles *A.arcuatus* sp. nov., but it can be distinguished by the following: 1) epigyne with a scape vs scape lacking (Fig. 1A, B); 2) copulatory openings on the posterior surface vs on the ventral surface (Fig. 1A, B); 3) median apophysis with 4 tapered tips vs 2 tapered tips (Fig. 2A, C, D); 4) tegular extension present vs lacking (Fig. 2); and 5) embolus longer than half the bulb diameter vs shorter than half the bulb diameter (Fig. 2D).

##### Description.

**Male** (holotype, Figs 16E, F, 17, 19K–N). Total length 2.40. Carapace 1.50 long, 1.25 wide. Abdomen 1.70 long, 1.30 wide. Clypeus 0.15 high. Eye sizes and interdistances: AME 0.10, ALE 0.08, PME 0.10, PLE 0.08, AME–AME 0.10, AME–ALE 0.13, PME–PME 0.10, PME–PLE 0.18, MOA length 0.30, anterior width 0.30, posterior width 0.28. Leg measurements: I 5.40 (1.70, 2.00, 1.25, 0.45), II 4.90 (1.50, 1.75, 1.20, 0.45), III 2.60 (0.85, 0.90, 0.50, 0.35), IV 3.85 (1.20, 1.30, 0.95, 0.40). Carapace pear-shaped, yellow, with sparse setae, cervical groove slightly distinct, inner base of PMEs brown. Chelicerae yellow, with 3 teeth on both margins. Endites yellow, tooth on anterior lateral edge, labium yellow. Sternum yellow, with sparse, dark setae. Legs yellow without annulus, tibia I with 9 macrosetae, tibia II with 10 macrosetae, tibia III with 6 macrosetae, tibia IV with 8 macrosetae. Abdomen slightly pointed anteriorly, blunt posteriorly, about 1.3 times longer than wide, dorsum whitish yellow with irregular grayish-yellow patch; venter grayish yellow, with sparse, dark setae. Spinnerets yellow.

***Palp*** (Fig. 17): with 1 patellar bristle; tegulum with tapered, apically pointed extension anterior to embolus; median apophysis prominent, with 4 protuberances; embolus thick at base, distal half slender and covered by edge of conductor; conductor longer than half of the bulb diameter, distally tapered into pointed tip.

**Female** (paratype IZCAS-Ar43135, Fig. 16A–D, G–H). Total length 3.50. Carapace 1.95 long, 1.35 wide. Abdomen 2.45 long, 1.95 wide. Clypeus 0.08 high. Eye sizes and interdistances: AME 0.10, ALE 0.08, PME 0.10, PLE 0.08, AME–AME 0.18, AME–ALE 0.18, PME–PME 0.13, PME–PLE 0.25, MOA length 0.30, anterior width 0.33, posterior width 0.30. Leg measurements: I 6.50 (2.00, 2.40, 1.55, 0.55), II 5.00 (1.80, 1.90, 0.85, 0.45), III 3.25 (1.20, 1.10, 0.60, 0.35), IV 4.40 (1.40, 1.50, 1.05, 0.45). Habitus similar to that of male except anterior abdomen much wider.

**Figure 18.**
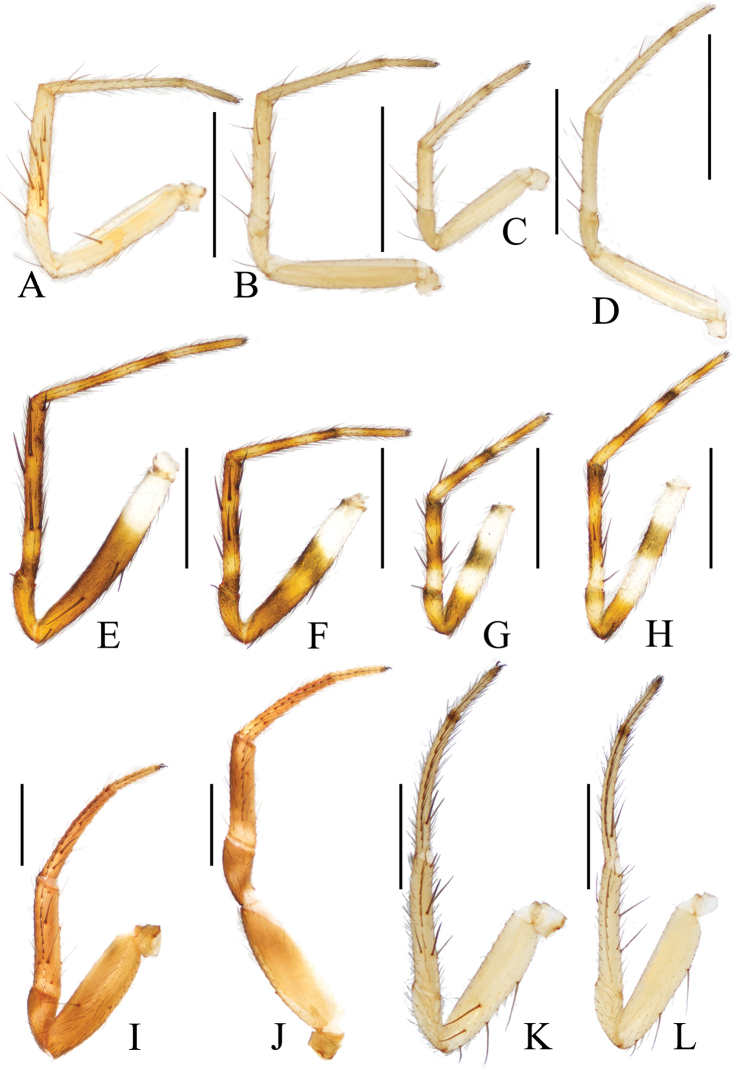
Legs of *Araneus* spp. **A–D***A.arcuatus* sp. nov., male holotype **A** leg I **B** leg II **C** leg III **D** leg IV **E–H***A.bidentatus* sp. nov., male holotype **E** leg I **F** leg II **G** leg III **H** leg IV **I, J***A.complanatus* sp. nov., female paratype Ar43106 **I** leg I **J** leg II **K, L***A.cucullatus* sp. nov., female paratype Ar43110 **K** leg I **L** leg II. Scale bars: 1 mm.

***Epigyne*** (Fig. 16A–D): slightly wider than long, with distally rimmed scape; copulatory openings depressed, posteriorly located; copulatory ducts slender, long, revolved about 360°; spermathecae spherical, touching each other.

**Figure 19.**
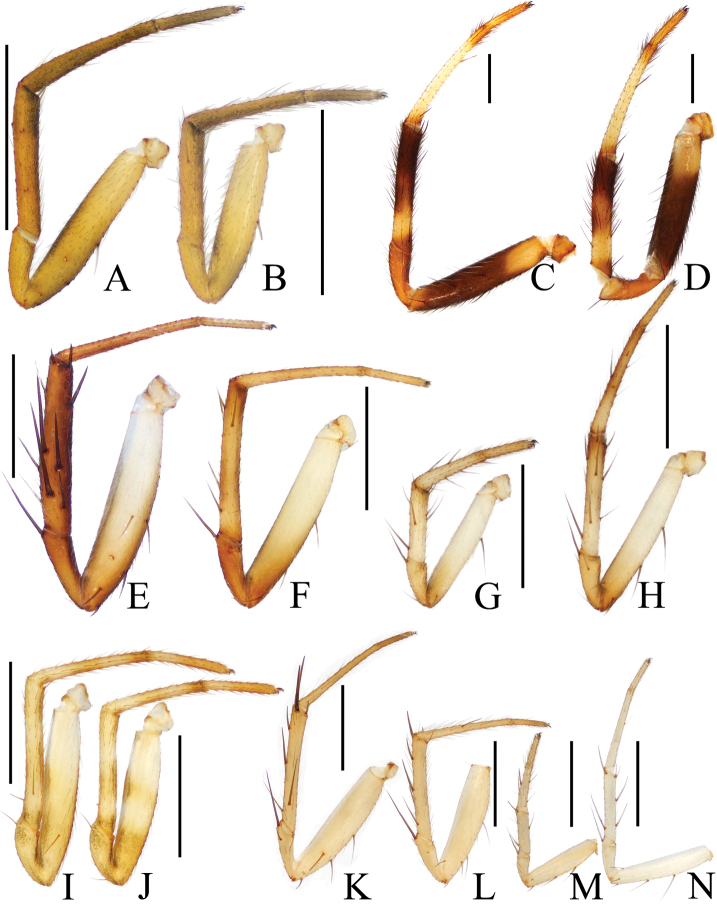
Legs of *Araneus* spp. **A, B***A.minisculus* sp. nov., male holotype **A** leg I **B** leg II **C, D***A.ovoideus* sp. nov., female holotype **C** leg I **D** leg II **E–H***A.pseudodigitatus* sp. nov., male holotype **E** leg I **F** leg II **G** leg III **H** leg IV **I, J***Araneussemiorbiculatus* sp. nov., female holotype **I** leg I **J** leg II **K–N***A.tetracanthus* sp. nov., male holotype **K** leg I **L** leg II **M** leg III **N** leg IV. Scale bars: 1 mm.

***Variation*.** Total length: ♂♂ 2.15–3.00; ♀♀ 3.15–4.00.

##### Distribution.

Known only from type localities (Yunnan, China).

## Supplementary Material

XML Treatment for Araneus

XML Treatment for Araneus arcuatus

XML Treatment for Araneus bidentatus

XML Treatment for Araneus bidentatoides

XML Treatment for Araneus complanatus

XML Treatment for Araneus corrugis

XML Treatment for Araneus cucullatus

XML Treatment for Araneus minisculus

XML Treatment for Araneus ovoideus

XML Treatment for Araneus pseudodigitatus

XML Treatment for Araneus semiorbiculatus

XML Treatment for Araneus tetracanthus
